# Metabolomics Profiles Reveal New Insights of Herpes Simplex Virus Type 1 Infection

**DOI:** 10.3390/ijms24021521

**Published:** 2023-01-12

**Authors:** Pu Huang, Xu Wang, Mengyue Lei, Ying Ma, Hongli Chen, Jing Sun, Yunzhang Hu, Jiandong Shi

**Affiliations:** 1 Yunnan Provincial Key Laboratory of Vector-Borne Diseases Control and Research, Institute of Medical Biology, Chinese Academy of Medical Sciences and Peking Union Medical College, Kunming 650118, China; 2 Institute of Medical Biology, Kunming Medical University, Kunming 650032, China

**Keywords:** herpes simplex virus type 1, metabolomics, host metabolism, viral replication

## Abstract

Herpes simplex virus type 1 (HSV-1) is a ubiquitous human pathogen that can cause significant morbidity, primarily facial cold sores and herpes simplex encephalitis. Previous studies have shown that a variety of viruses can reprogram the metabolic profiles of host cells to facilitate self-replication. In order to further elucidate the metabolic interactions between the host cell and HSV-1, we used liquid chromatography-tandem mass spectrometry (LC-MS/MS) to analyze the metabolic profiles in human lung fibroblasts KMB17 infected with HSV-1. The results showed that 654 and 474 differential metabolites were identified in positive and negative ion modes, respectively, and 169 and 114 metabolic pathways that might be altered were screened. These altered metabolites are mainly involved in central carbon metabolism, choline metabolism, amino acid metabolism, purine and pyrimidine metabolism, cholesterol metabolism, bile secretion, and prolactin signaling pathway. Further, we confirmed that the addition of tryptophan metabolite kynurenine promotes HSV-1 replication, and the addition of 25-Hydroxycholesterol inhibits viral replication. Significantly, HSV-1 replication was obviously enhanced in the ChOKα (a choline metabolic rate-limiting enzyme) deficient mouse macrophages. These results indicated that HSV-1 induces the metabolic reprogramming of host cells to promote or resist viral replication. Taken together, these observations highlighted the significance of host cell metabolism in HSV-1 replication, which would help to clarify the pathogenesis of HSV-1 and identify new anti-HSV-1 therapeutic targets.

## 1. Introduction

Herpes simplex virus (HSV) is a common pathogen found in the natural human host, belonging to the *Alphaherpesvirinae* subfamily of *Herpesviridae*. HSV-1 is an enveloped double-stranded DNA virus with a 152-kbp genome and more than 80 different open-reading frames [1]. The virion of HSV-1 is a spherical particle with a diameter of 186 nm, and the protruding glycoproteins of the virion make its full diameter approximately 225 nm [2]. HSV-1 is highly contagious, spreading through contact with the pathogens via the oral mucosa or ocular mucosa. The virus can also establish a latent infection in the human body and is still infectious. According to the epidemiological investigation, about 118 million new cases of HSV-1 infection were recorded in the world in 2012, and the global prevalence rate was about 90% [3]. Approximately 50% of children and 70% of adults were infected in Asia in 2019, with the majority of infections occurring in childhood [4]. Herpetic gingival stomatitis is the most common form of primary herpes simplex, which often occurs in children. HSV-1 infection can also cause neurological diseases, such as facial paralysis, vestibular neuritis, and herpes simplex encephalitis. In the brain, the site of the HSV-1 infection and the central nervous system affected by Alzheimer’s disease are located in the same region, and epidemiological and experimental data suggest that HSV-1 is involved in the pathogenesis of the disease [5]. HSV-1 can cause herpes simplex pneumonia [6], severe respiratory diseases, and concurrent infections in patients with compromised immune systems (such as malnutrition, malignancies, burns, and severe lung diseases) [7]. Consequently, the global healthcare burden of diseases caused by HSV-1 is significant. Nevertheless, there is no vaccine against HSV-1 on the market, and the treatment of HSV infection mainly relies on acyclovir (ACV) and penciclovir (PCV) and their respective prodrugs: valacyclovir and famciclovir. The use of ACV leads to the development of drug resistance in HSV-1, with a high rate of drug resistance in immunocompromised patients [8]. Therefore, understanding the mechanism of HSV-1 and screening new antiviral targets to develop new drugs is essential.

In nature, all viruses cannot replicate independently to produce offspring, and their life cycle is completely dependent on the host metabolism. After invading the host cells, the virus can significantly alter the cell’s metabolism [9]. Thus, it is necessary to determine how a cell’s metabolism is altered after viral infection, which would provide insight into the requirements of viral replication and reveal potential targets for viral inhibition [10]. The first metabolomics study in the field of virology was reported by Munger et al., who measured the levels of 63 different intracellular metabolites during HCMV infection using liquid chromatography-tandem mass spectrometry (LC-MS/MS). The study suggested that HCMV enhances glycolytic flux and carbon transport from glucose to the tricarboxylic acid cycle (TCA) to promote fatty acid biosynthesis. In addition, TOFA is an inhibitor of fatty acid synthesis and elongation and can be used to target HCMV and inhibit its replication [11]. In 2011, Vastag et al. compared the metabolic characteristics of HSV1-infected cells with HCMV [12]. Moreover, several viruses have been shown to alter multiple metabolic pathways in cells and expand the number of cellular metabolites [13,14,15,16,17]. For example, hepatitis C virus infection dysregulates glucose utilization and lipid biosynthesis [18]. Rhinovirus-infected cells rapidly regulate uptake in a PI3K-dependent manner and enhance the expression of PI3K-regulated glucose transporter GLUT1, revealing the critical role of glucose metabolism in viral replication [19]. Kaposi’s sarcoma-associated herpesvirus (KSHV) upregulates fatty acid synthesis, and the fatty acid synthesis inhibitors lead to the death of latently infected KSHV-infected cells, thus providing novel therapeutic targets for KSHV and ultimately Kaposi’s sarcoma tumors [20]. Based on the above results, the changes in metabolites and metabolic pathways reflect the virus-mediated regulation of the host metabolic pathways to facilitate or restrict self-replication of the virus. This is also beneficial in understanding the changes in metabolic pathways adopted by the host cells to resist virus infection.

Taken together, researchers will focus not only on the changes in a few metabolites but will also build a comprehensive profile of all metabolites after a virus infects a cell. Metabolomics is a method that has been developed and applied to measure the abundance of all metabolites in cells and organisms, thereby allowing a comprehensive quantitative analysis of these molecules in biological systems [21]. Because of its high sensitivity and specificity, metabolomics is considered an effective tool for exploring the interactions between human organisms and viruses [22]. Metabolomics was first introduced in 1999. Nicholson et al. analyzed the metabolic response of living systems to pathophysiological stimuli through a multivariate statistical analysis of biological and nuclear magnetic resonance spectral data [23]. Since then, metabolomics has been widely used in gene function, toxicology, plant science, environmental analysis, clinical diagnosis, and nutritional and biological genotype identification studies [24].

Human embryonic lung diploid cells (KMB17) were derived from the normal lung tissue of a 4-week-old female embryo, and a cell line was established in 1975 at the Kunming Institute of Medical Biology, Chinese Academy of Medical Sciences. It has no carcinogenicity or contamination by exogenous factors but has stable cell characteristics and sensitivity to several viruses; examples include Coxsackievirus [25], hepatitis A virus [26] and herpes simplex virus [27]. It can be used as a model to study the interaction between viruses and human cells [28]. To elucidate the comprehensive metabolic changes of HSV-1 acutely infected human fibroblasts in vitro, we conducted an untargeted metabolomic analysis to analyze the metabolic profiles of human lung fibroblasts KMB17 infected with HSV-1. The results showed that HSV-1 remodels the metabolic networks in KMB17 cells with respect to the changes in the central carbon metabolism. These observations highlighted the significance of the host cell metabolism in HSV-1 replication, suggesting that HSV-1 induces the metabolic reprogramming of host cells to promote or resist viral replication. Our findings would help to clarify the pathogenesis of HSV-1 and reveal new anti-HSV-1 therapeutic targets.

## 2. Results 

### 2.1. HSV-1 Infects KMB17 Cells

**Figure 1:**
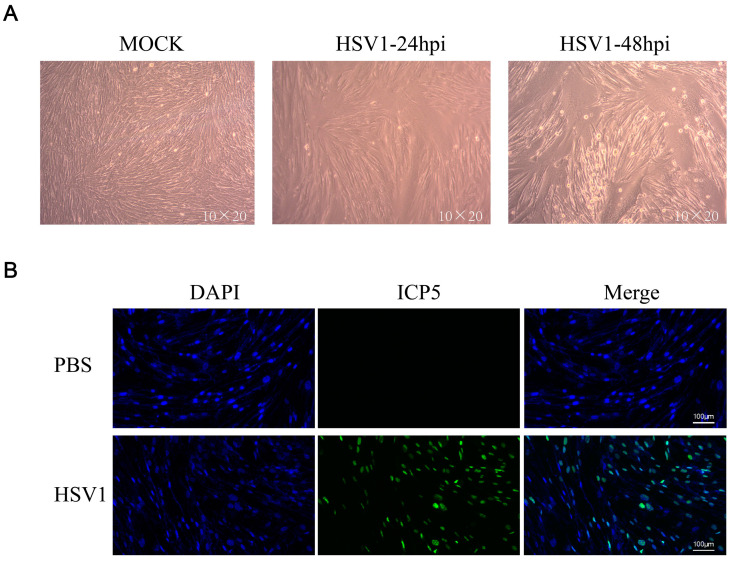
HSV-1 effectively infected KMB17 cells. (**A**) KMB17 was infected with HSV-1 (MOI = 0.1), cultured in 2% serum maintenance solution for 24 or 48 h, and recorded under microscope (10 × 20). In MOCK group, PBS was used as the control. After 48 h of incubation under the same conditions, the samples were observed and recorded under the microscope. (**B**) KMB17 cells were infected with HSV-1 (MOI = 0.1) and cultured in 2% serum maintenance solution for 24 h before immunofluorescence. Then, images were observed and collected under a fluorescence microscope. Blue is DAPI, and green is HSV1 virus late protein (ICP5).

KMB17 cells were infected with the HSV-1 virus (MOI = 0.1) and observed under a microscope after 24 or 48 h. The results showed that 24 h post-HSV1 infection, the cell morphology changed slightly compared to the control group, and 48 h after HSV1 infection, KMB17 cells showed obvious cytopathic effects, while the PBS control cells grew adequately (Figure 1a). In addition, KMB17 cells were infected with the HSV-1 virus (MOI = 0.1), and the replication and proliferation of the virus were detected by immunofluorescence. However, after 24 h, no fluorescence signal was detected in the control group, while about 70% of cells in the HSV-1 infection group exhibited the fluorescence signal (Figure 1b). These results indicated that HSV-1 infected, replicated, and proliferated in KMB17 cells. The results suggested that KMB17 cells could be infected with the HSV-1 virus to prepare metabolome sequencing samples; subsequently, the changes in host cell metabolites were analyzed. An MOI of 0.1 was selected to collect the samples in 24 h according to the condition that the virus did not cause obvious pathological changes in the host cells after infection and that the virus could infect at least 50% of the cells.

### 2.2. Reliability Assessment of LC-MS Data

**Figure 2:**
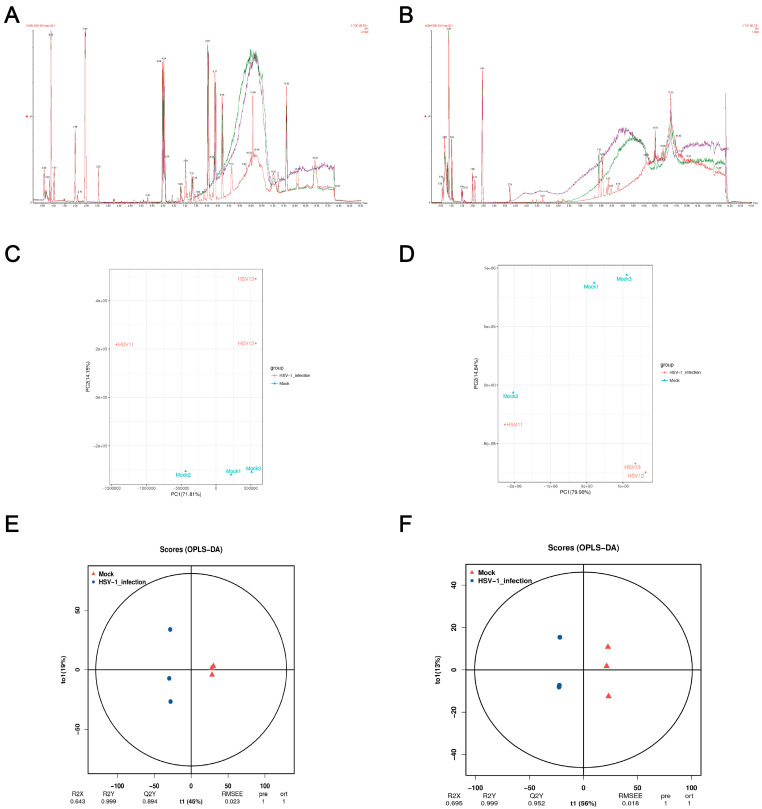
Quality control (QC) and multivariate statistical analysis. (**A**,**B**) Total ion BPI overlap plots of QC samples in positive and negative ion modes. (**C**,**D**) Principal component analysis diagram of difference grouping under positive and negative ion mode. Where the X-axis represents the first principal component, and the Y-axis represents the second principal component. Each point in the figure represents a sample. Samples in the same group are represented with the same color, and samples in different groups are marked with different colors. (**E**,**F**) OPLS-DA score in positive and negative ion mode. The X-axis (t1) represents the predictive component (inter-group difference component), the Y-axis (t2) represents the orthogonal component (intra-group difference component), and the percentage of the transverse Y-axis represents the proportion of the component in the total variance. At the bottom of the figure are the parameters of the model, including R2X, R2Y, Q2Y, RMSEE (root mean square error), pre (number of predicted components), and ort (number of orthogonal components).

Monitoring the stability of the instrument and whether the signal is normal in real time during the detection process is essential. The BPI peak retention time and peak area of the QC sample overlapped (Figure 2a,b), indicating that the analysis system had a good signal, high stability, and repeatability. In order to understand the overall metabolic differences between the samples in each group and the degree of variation between the samples within the group, we performed a PCA on the samples. The PCA score plot showed significant separation between the control and the HSV1-infected groups in the positive ion mode, and the samples within the group were clustered (Figure 2c,d). In the negative ion mode, except Mock2 and HSV1-1, all the samples showed satisfactory intergroup separation and intragroup aggregation. These results indicated that the differences between the samples were large, the repeatability of the samples within the group was good, and the differential metabolites obtained by subsequent difference analysis were reliable. Although the PCA method extracted the main information, it is not sensitive enough for the variables with a small correlation. The orthogonal projections to latent structures-discriminant analysis (OPLS-DA) method can resolve this issue. The prediction parameters to evaluate this model are based on R2Y and Q2Y, and the closer the two values are to one, the more stable and reliable the model is. Interestingly, Q2Y > 0.5 is considered an effective model, and Q2Y > 0.9 is an excellent model, which can be analyzed and screened according to VIP. A clear separation between the control and HSV1-infected groups was observed in the OPLS-DA score plot, indicating that the two groups had different metabolomic characteristics. In the positive ion mode, R2Y and Q2Y were 0.999 and 0.894, respectively. In the negative ion mode, R2Y and Q2Y were 0.999 and 0.952, respectively. This indicated the stability and reliability of the OPLS-DA model (Figure 2e,f), which could be used to screen and analyze differential metabolites according to VIP.

### 2.3. Total Metabolite Annotation Results

**Figure 3:**
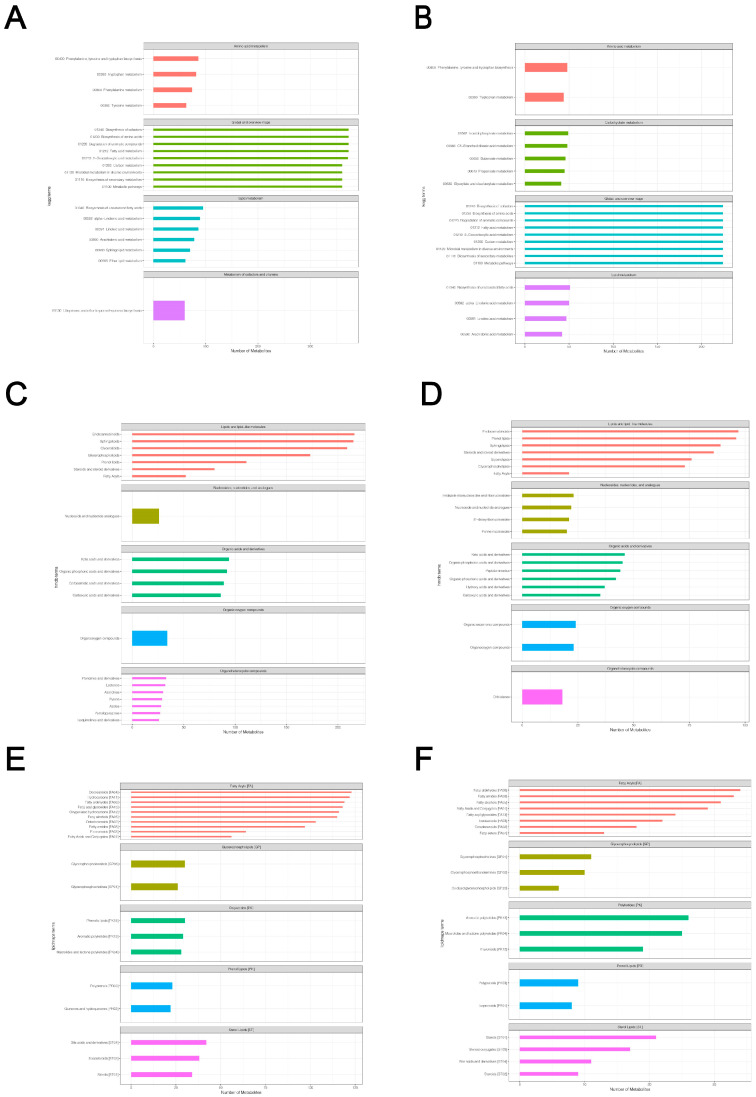
Total metabolite annotation results. (**A**,**B**) Annotation results of KEGG database in positive and negative ion mode. The entries under the same box in the figure represent the hierarchical classification annotation of KEGG pathways, corresponding to KO Pathway Level 1 and KO Pathway Level 2. (**C**,**D**) Annotation result of HMDB database in positive and negative ion mode. The entries under the same box in the figure represent HMDB level classification information, corresponding to the superclass and class information of HMDB database. (**E**,**F**) Lipid-MAPS database annotation results in positive and negative ion mode. The entries in the same box in the figure represent HMDB hierarchy classification information, which corresponds to CATEGORY and MAIN-CLASS information of the LIPID MAPS database. Column length represents the number of metabolites annotated for the classification.

Based on the LC-QTOF platform, we performed a qualitative and quantitative metabolome analysis on six HSV1-infected and uninfected samples and identified 7727 peaks in the positive ion mode; of these, 2196 metabolites were annotated (Supplementary File S1). A total of 4747 peaks were detected in the negative ion mode, and 1066 metabolites were annotated (Supplementary File S2). The KEGG database was used to annotate all the identified metabolites. The top 20 most annotated metabolites under the KO pathway level 2 entry were selected to draw a summary bar chart (Figure 3a,b). In addition, the Human Metabolome Database (HMDB) and LIPID Metabolites and Pathways Strategy (LIPS-MAPS) were used to annotate the detected metabolites. The top 20 annotated metabolites with the largest number were selected for the construct (Figure 3c–f). The metabolic processes involved were: amino acid metabolism, carbohydrate metabolism, cofactors biosynthesis, aromatic compound degradation, lipid and lipid-like molecules metabolism, nucleotide and its analogs metabolism, organic acids and their derivatives metabolism, and secondary metabolites biosynthesis. A large number of metabolites and the abundance of metabolic pathways open several possibilities for subsequent screening and identification of differential metabolites.

### 2.4. Screening of Differential Metabolites

**Figure 4:**
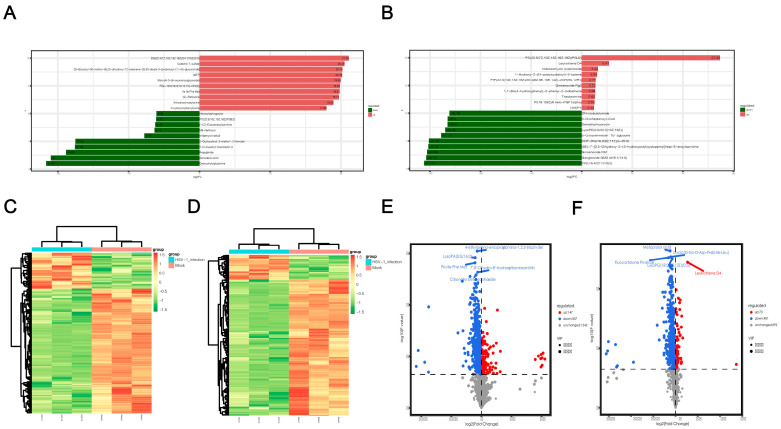
Screening results of differential metabolites. (**A**,**B**) Histogram of differential multiples of metabolites in positive and negative ion mode. The label of each column indicates the metabolite name, red for up-regulation, green for down-regulation, and column length for logFC. The figure shows the top 10 substances in logFC order. (**C**,**D**) Clustering heat map of differential metabolites in positive and negative ion mode. X-axis is each sample, Y-axis is the quantitative value of metabolite z-score standardized after hierarchical clustering, and the color bar on the top distinguishes different groups. (**E**,**F**) Volcano map of differential metabolites in positive and negative ion mode. Each point on the volcano plot represents a metabolite, the X-axis represents the fold change (take the logarithm base 2), and the Y-axis represents the *p* value of the *t*-test (take the logarithm base 10). Blue dots in the figure represent downregulated differentially expressed metabolites, red dots represent upregulated differentially expressed metabolites, and gray represents metabolites detected but not significantly different. In addition, the first five qualitative metabolites were selected and labeled in the figure after sorting by *p* value.

After a qualitative and quantitative analysis of the detected metabolites, we used a univariate statistical method to analyze the FC of the quantitative information of the metabolites in each group (Supplementary Files S3 and S4). The top 10 metabolites were upregulated and downregulated, respectively, in the experimental group compared to the control group (Figure 4a,b). LogFC results showed that the top 10 significantly up- or downregulated differential metabolites detected in positive and negative ion modes were significantly different, which could be attributed to the large total amount of differential metabolites and the difference in electrodes. In the positive ion mode, the metabolites significantly upregulated after HSV1 infection included catechin 7-sulfate and malvidin 3-(6-coumaroyigiucoside), and the substances significantly downregulated included deoxycholylglutamine and repaglinide. In the negative ion mode, the metabolites significantly upregulated after HSV1 infection were PS and leukotriene D4, and those significantly downregulated included PC and ganglioside GM3. Metabolomics data are characterized by high dimensionality and mass. Thus, it is necessary to combine the multivariate statistical analysis methods and analyze the differential metabolites from multiple perspectives according to the characteristics of the data to determine the differential metabolites accurately. Therefore, the VIP of the OPLS-DA model and *p* value or the FC of the univariate analysis were used to further screen the differential metabolites. Screening conditions were: VIP > 1 and *p* value < 0.05. In the positive ion mode, 654 metabolites with significant differences were screened from 2196 total metabolites. Compared to the control group, 147 metabolites were significantly upregulated, and 507 metabolites were significantly downregulated in the HSV1-infected group (Supplementary File S5). In the negative ion mode, 474 metabolites with significant differences were screened from 1066 total metabolic species, including 73 significantly upregulated and 401 significantly downregulated metabolites (Supplementary File S6). The top 10 differential metabolites in the positive and negative ion modes, respectively, are shown here in descending order of VIP values (Table 1). Hierarchical clustering heat maps were drawn based on the abundance patterns of the overall differential metabolites. The heat map showed a significant difference in metabolite abundance between the HSV1-infected and uninfected groups (Figure 4c,d). In order to view the overall difference in trend and statistical significance of metabolites in the two groups, we plotted a volcano plot of differentially expressed metabolites. The volcanic map showed that KMB17 cells infected with HSV1 had significant changes in more metabolites, and the significantly downregulated metabolites were more abundant than the significantly upregulated metabolites (Figure 4e,f). These results suggest that the HSV1 virus infection of the KMB17 cells alters the metabolites produced by the host cells.

### 2.5. Differential Metabolite Annotation and Pathway Enrichment Analysis

**Figure 5:**
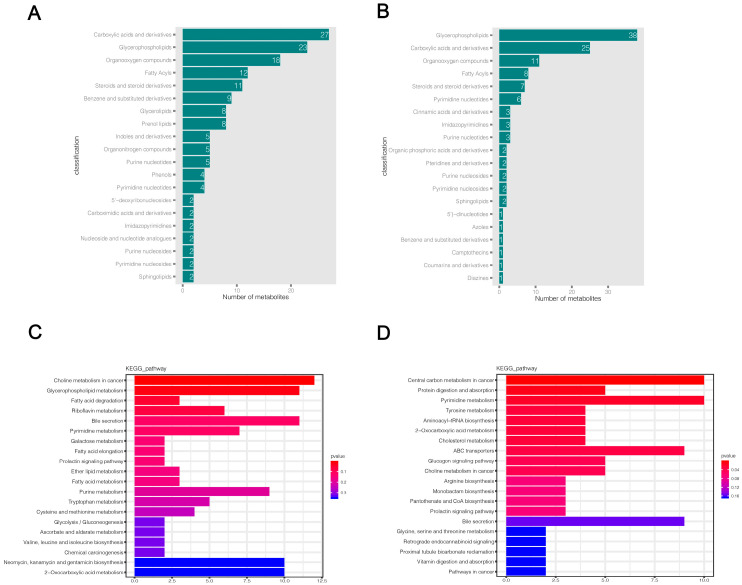
Significantly different metabolites annotation and KEGG metabolic pathway enrichment analysis. (**A**,**B**) Annotation results of HMDB database for differential metabolites. Each column represents the number of substances annotated in an HMDB classification. (**C**,**D**) KEGG functional annotation and enrichment analysis of differential metabolites. The X-axis is the number of differential metabolites annotated to the pathway, and the Y-axis is the pathway name. The color depth represents the enrichment degree, and the redder the enrichment, the more significant.

HMDB and KEGG databases were used to annotate the final selected differential metabolites. The HMDB classification map only shows the top 20 classification entries (Figure 5a,b). The HMDB annotation results showed that a large number of differential metabolites were enriched in carboxylic acids and derivatives, glycerophospholipids, organic compounds, fatty acyls, steroids, and steroid derivatives in both positive and negative ion modes, while a small number of differential metabolites were distributed in benzene and substituted derivatives, pyrimidine nucleotides, purine nucleotides, and other metabolic categories (Supplementary Files S7 and S8). The results of the KEGG functional annotation of differential metabolites are shown in Supplementary Files S9 and S10. Then, we used the hypergeometric test method of clusterProfiler to enrich and analyze the annotation results of KEGG for differential metabolites. We found that 169 and 114 pathways were altered in KMB17 cells infected with HSV1 in positive and negative ion modes, respectively (Supplementary Files S11 and S12). The top 20 metabolic pathways with the highest significance were selected according to the *p* value and presented as histograms (Figure 5c,d). The metabolic pathways significantly altered in the positive ion mode include choline metabolism in cancer, glycerophospholipid metabolism, fatty acid degradation, riboflavin metabolism, pyrimidine metabolism, bile secretion, prolactin signaling pathway, galactose metabolism, glycolysis and gluconeogenesis, purine metabolism, and multiple amino acid metabolism pathways: for example, tryptophan, cysteine, methionine metabolism and valine, leucine, isoleucine biosynthesis. These results suggested a significant correlation between these pathways and the response of HSV1 to KMB17 infection. In the negative ion mode, central carbon metabolism in cancer, protein digestion and absorption, pyrimidine metabolism, choline metabolism in cancer, pantothenate and CoA biosynthesis, prolactin signaling pathways, bile secretion, and various amino acid metabolic pathways (including tyrosine metabolism, ABC transporters, arginine biosynthesis, hydrogen acid, silk hydrogen acid, and Sue hydrogen acid metabolism) are closely related to HSV1 infection. We found that choline metabolism in cancer, bile secretion, prolactin signaling pathway, pyrimidine metabolism, 2-carboxylic acid metabolism, and various amino acid metabolism pathways are significantly enriched in both positive and negative ion modes. The analysis of metabolic pathways showed that the metabolite profile of KMB17 cells was reprogrammed after HSV1 infection.

### 2.6. Effects of Several Different Metabolites and Metabolic Pathways on Virus Replication

**Figure 6:**
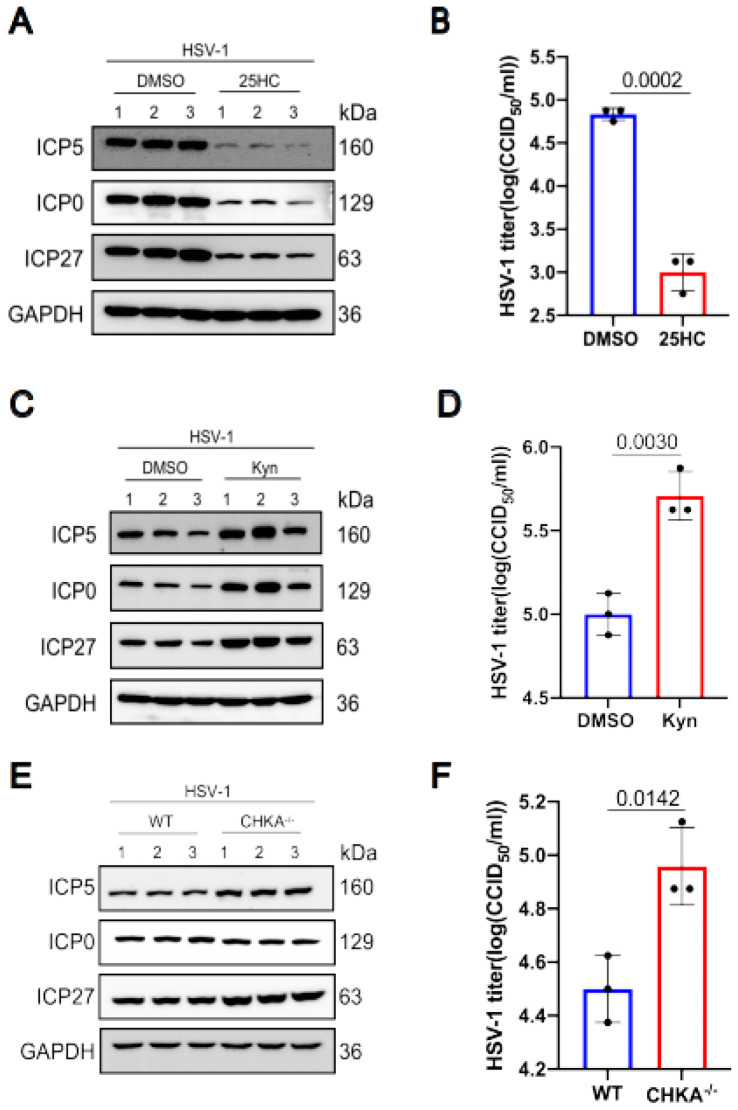
The effects of 25-Hydroxycholesterol, choline kinase and kynurenines on HSV1 replication. KMB17 cells were pretreated with 5 μM 25-Hydroxycholesterol and DMSO of the same volume for 24 h and then infected with HSV-1 (MOI = 1). After 12 h, the total protein was collected for Western blot to detect the viral proteins ICP0, ICP5, ICP27 and the host protein GAPDH (**A**). The supernatant was collected 12 h later, and the virus titer was determined by CCID50 method (**B**). KMB17 cells were pretreated with 400 μM kynurenines and equal volume DMSO for 24 h and then infected with HSV-1 (MOI = 1). After 24 h, total proteins were collected for Western blot to detect viral proteins ICP0, ICP5, ICP27 and host protein GAPDH (**C**). The supernatant was collected 24 h later, and the virus titer was determined by CCID50 method (**D**). Wild-type and CHKA gene knockout RAW264.7 cells were infected with HSV-1 (MOI = 1). After 12 h, the total protein was collected for Western blot to detect the viral proteins ICP0, ICP5, ICP27 and the host protein GAPDH (**E**). The supernatant was collected 12 h later, and the virus titer was determined by CCID50 method (**F**). Data are presented from at least three independent experiments (mean ± SD). *p* values were determined using a two-tailed, unpaired Student’s *t*-test.

We selected three differential metabolites to analyze their influence on HSV-1 replication. The results showed that some differential metabolites promote virus replication while others inhibit virus replication. The metabolite 25-Hydroxycholesterol decreased after HSV-1 infection. So we pretreated KMB17 cells with 25-Hydroxycholesterol for 24 h, and then infected them with the HSV-1 virus. As shown in Figure 6a,b, the viral protein expression in the 25-Hydroxycholesterol pretreated group significantly decreased compared with the control group, and the virus titer also decreased in the 25-Hydroxycholesterol pretreated group. These results suggested that 25-Hydroxycholesterol inhibits HSV-1 replication. In HSV-1 infected KMB17 cells, tryptophan is obviously down-regulated. Kynurenine is one of the most important metabolites of tryptophan. To analyze the effect of kynurenine on HSV-1 replication, KMB17 cells were pretreated with kynurenine and followed by infection with HSV-1. The results showed that the viral protein expression and viral titer significantly increased in the group treated with kynurenine compared with the control group, as shown in Figure 6c,d, indicating that HSV-1 enhanced its own replication by promoting host cell tryptophan metabolism. Choline metabolism was enhanced during HSV-1 infection. Choline metabolism requires the participation of choline kinase, so we knocked out the choline kinase gene of RAW264.7 cells, and then the choline kinase knocked out the cells, and wild-type cells were infected with HSV-1. The results showed that the expression of HSV-1 virus protein increased, and the virus titer also increased significantly in the choline kinase gene knockout cell line compared to the wild-type cell line, as shown in Figure 6e,f. These results suggested that the accumulation of choline could promote virus replication. However, after HSV-1 infected KMB17 cells, choline was significantly down-regulated, indicating that in the process of virus infection, host cells actively regulated choline metabolism to inhibit virus replication. It is worth noting that not every differential metabolite plays a role in virus replication and proliferation, but as long as we can screen out the key differential metabolite that affects virus replication, it is promising to develop new drugs based on this target to fight virus infection.

## 3. Discussion 

Viruses are the most abundant and widely distributed biological entities worldwide, with varied genetic material and the ability to infect different species [29]. Furthermore, their lives depend on their host’s metabolism, including virion uncoating, genome replication, and progeny packaging [9]. The main purpose of the host’s metabolism is to convert nutrients into energy to sustain all cellular processes and provide raw materials for the biosynthesis of proteins, lipids, and nucleic acids. Therefore, a reciprocal correlation was established between virus replication and host defense. Viruses have developed mechanisms to promote self-replication by altering key host metabolic pathways and major regulatory proteins that target the metabolism. Accordingly, hosts can also alter the original metabolic pathways to contain the virus infection [30], and metabolomics provides insight into tracing such checkpoints [31]. In recent years, many viruses have been shown to alter the metabolism of infected cells [32]. The present study used the LC-MS/MS method to analyze the comparative metabolomic characteristics between HSV1-infected and uninfected KMB17 cells. These findings provide new insights into the response of KMB17 to HSV1 in order to define the mechanism of infection.

For HSV1 virus infection, a high MOI or a long infection time could induce strong lysis of KMB17 cells. In order to not disturb the cell structure after HSV1 infection and cause more significant changes in host metabolites, we selected an MOI of 0.1 and collected samples in 24 h. These samples were subjected to metabolomic analysis using LC-MS/MS technology. The results showed that approximately 500 significantly different samples were found in positive and negative modes. Additionally, 169 and 114 pathways were identified in the positive and negative modes of KMB17 cells infected with HSV1, respectively, after an enrichment analysis of the annotation results of KEGG. Furthermore, we described several metabolic pathways with highly significant differences, enriched in both positive and negative ions.

Strikingly, central carbon metabolism plays a crucial role in many cellular biological processes, which are the main sources of energy required by organisms, providing precursors for other metabolisms in the body [33]. Several studies have reported that different types of viruses can reshape host metabolic networks after infecting host cells. These findings also seem to have a common feature: viruses preferentially manipulate central carbon metabolism pathways to increase available energy by altering glycolysis and glutamine metabolism [34]. In the present study, the top 20 metabolic pathways detected after HSV1 infection in KMB17 cells included the glycolysis pathway, an evolutionarily conserved and oxygen-independent process to generate ATP and pyruvate. After glycolysis, pyruvate is oxidized to acetyl-CoA in the presence of oxygen and used in the tricarboxylic acid (TCA) cycle, in which hydrocarbons can be completely burned and metabolized to CO_2_ and water, providing the energy needed for viral replication.

In addition to energy, viral replication requires the synthesis of nucleic acids, amino acids, and complex lipids. Thus, the virus must actively transfer some hydrocarbons from complete combustion in the TCA cycle into the anabolic pathways. In 2011, a study mainly analyzed the difference in metabolism between HCMV and HSV-1 and emphasized the regulation of the TCA cycle to promote HSV-1 to produce pyrimidine or HCMV to synthesize fatty acids^12^. Our results are consistent with this. The results showed that in HSV-1 infected and uninfected cells, the fatty acid synthesis pathway did not change significantly, but the metabolism of pyrimidine and purine showed significant differences. Previous studies focused on the changes in pyrimidine after HSV-1 infection, providing a strong basis for the treatment of HSV-1-induced diseases by nucleoside nucleotide analogues. Nonetheless, this therapeutic mechanism provides a reference for inhibiting other viral infections because almost all viruses need to synthesize pyrimidines or purines to complete viral replication while infecting the body. For viruses that need to synthesize purines and pyrimidines in large quantities in the early stage, targeting pyrimidine or purine biosynthetic pathways is a promising approach to inhibit viral replication. A broad-spectrum antiviral drug, compound A3, has been found to interfere with de novo pyrimidine synthesis. A previous study demonstrated that A3 acts by depleting the pyrimidine reservoir, the key to effective viral replication, and has a broad spectrum of antiviral effects [35]. In addition, targeting pyrimidine biosynthesis inhibits enterovirus replication [36]. These findings are encouraging, but the main anti-herpes drugs, including acyclovir and valaciclovir, exhibit drug resistance. Therefore, this study is committed to guiding researchers to explore and discover new targets for anti-HSV-1 therapy. Our results also showed significant changes in choline metabolism, cholesterol metabolism, bile secretion, prolactin signaling pathway, and glucagon signaling pathway after HSV-1 infection in KMB17 cells. Intriguingly, both cholesterol metabolism and bile acid metabolism are related to innate antiviral immunity [37,38], and choline metabolism is related to HCV replication [39]. This finding suggested a similar role of these metabolites in anti-HSV-1 infection. Thus, we explored the effects of choline and cholesterol metabolism on HSV-1 replication. The results showed that 25-Hydroxycholesterol inhibits HSV-1 replication, while attenuated choline metabolism promotes virus replication. Although their mechanisms are not fully understood, these results show that it is possible to develop new drugs against HSV-1 based on these targets. Amino acids are crucial for living organisms. Many metabolomic studies have identified metabolic changes in amino acids caused by viral infections [9,40,41]. For example, murine norovirus controls the antiviral response by activating metabolic stress responses, which activate amino acid responses and impair inflammatory signaling [42]. In the present study, we obtained similar results. Moreover, significant changes in amino acid metabolic pathways were observed after HSV1 infection in KMB17 cells, including the synthesis of arginine, valine, leucine, and isoleucine. The increase in the amino acid pool may contribute to the rapid proliferation of viral protein synthesis and virion assembly. In addition, we focused on tryptophan metabolism. After the HSV1 infection of KMB17 cells, the tryptophan metabolic pathway was altered significantly, and the tryptophan content was significantly reduced. Tryptophan is essential in various metabolic pathways and a major component of protein synthesis and the generation of several biomolecules, such as kynurenine [43]. In this study, KMB17 cells were pretreated with kynurenine and followed by infection with HSV-1. The results showed that the expression of viral proteins significantly increased, indicating that the virus enhanced its replication by promoting tryptophan metabolism. Tryptophan degradation is initiated by indoleamine-2,3-dioxygenase (IDO1), which was identified for its potent anti-HIV2 activity in a comprehensive screening of antiretroviral IFN-stimulated genes [44]. Subsequently, the present study confirmed that IDO1 reduces viral production by >100-fold due to severe inhibition of viral protein synthesis. Supplementation with L-tryptophan or its competitive inhibitor 1-methyl-L-tryptophan (1-MT) may rescue HIV production [45], thereby supporting the theory that the antiviral effect of IDO1 is driven by tryptophan depletion. Since then, IDO1 has been shown to inhibit the growth of several viruses, including HSV2 [46], vaccinia virus [47], HCV [48], IAV [49], parainfluenza virus (PIV3) [50], and HBV [51]. Although the mechanism of IDO1 in HSV1 infection has not been reported, the results of this study showed that the host could target its own tryptophan metabolism after HSV1 infection with KMB17, suggesting IDO1 as a potential target for anti-HSV1 therapy.

In August of this year, it was reported that the HSV1 virus in vivo infected the Murine trigeminal ganglia and reshaped the host’s metabolic network [52], thereby limiting viral replication. The differential metabolites and pathways identified in this report are similar to the in vitro screening results after HSV1 virus infection with KMB17, especially amino acid metabolism and nucleotides. Moreover, this fully shows that HSV1 has consistent metabolic regulation on the host after in vitro or in vivo infection of the host. This is of great significance for the later screening of antiviral targets and drugs. In addition, compared with the description of the metabolic profile of HSV1 in vitro infected cells in 2011 [12], we screened more differential metabolites, providing more possible antiviral targets. In addition, the strains and cells we used were also different from those before, but the significantly different metabolites obtained had high coincidence, which indicated that the host metabolism changes caused by different strains of HSV1 after infecting different cells were relatively conservative.

## 4. Materials and Methods

### 4.1. Cells and Virus

KMB17 cells and Vero cells were cryopreserved in our laboratory and revived from liquid nitrogen. A CHKA-deficient RAW264.7 cell line was generated by CRISPR/Cas9-mediated genome engineering (HanBio Company, Shanghai, China). Wild-type RAW264.7 cells were also provided by HanBio Company. All cells were cultured in Dulbecco’s modified Eagle’s medium (DMEM) at 37 °C with 5% CO_2_, supplemented with 10% fetal bovine serum, penicillin (100 U/mL), and streptomycin (100 µg/mL). Additionally, HSV-1 strain 17 (Gene Bank access No. NC_001806.2) was amplified and titrated in Vero cells.

### 4.2. Immunofluorescence Assay

The cells were seeded in six-well plates with 1.5 × 10^6^ cells per well and cultured overnight. The original medium was removed the following day and incubated with serum-free medium and the HSV1 virus (multiplicity of infection (MOI) = 0.1) for 1 h. Then, the medium was removed, the cells were washed twice with phosphate-buffered saline (PBS), and a maintenance solution (with 2% fetal bovine serum) was added to continue the culture. After 24 h, the cells were collected, fixed with 4% paraformaldehyde for 30 min, permeabilized with 0.5% Triton X-100 at room temperature for 20 min, and blocked with 5% bovine serum albumin (BSA) for 30 min. Then, each slide was incubated with diluted primary antibody ICP5 (Abcam, Cambridge, UK; 1:500) in a humidified chamber box overnight at 4 °C. Subsequently, the slides were washed with PBST and incubated with FITC-labeled fluorescent secondary antibody (Proteintech, Chicago, CA, USA; 1:200) at 37 °C for 1 h. Finally, the slides were sealed by dropping the sealing liquid containing DAPI and an anti-fluorescence quencher, and the images were observed and collected using a panoramic MIDI digital scanner (3D HISTECH, Budapest, Hungary), as described previously [53].

### 4.3. Sample Preparation

KMB17 cells were cultured in six T75 culture flasks at 37 °C with 2 × 10^7^ cells/flask. At 90% confluency, the monolayers were inoculated with HSV-1 virus (MOI = 0.1) in three of the culture flaps, and the other three were used as uninfected controls, providing three HSV-1-infected samples and three uninfected samples. After 24 h, HSV-1 and uninfected supernatants were removed, and the cells were harvested by trypsin digestion and centrifugation at 1000 rpm for 1 min on a precooled rotor at 4 °C. The supernatant was discarded, and the cells were frozen in liquid nitrogen for 30 s and stored at −80 °C.

### 4.4. Metabolite Extraction and Liquid Chromatography Separation

Cells were lysed with metabolite extract (volume ratio of methanol to acetonitrile = 1:1, internal standard concentration 2 mg/L), vortexed, sonicated in an ice-water bath for 10 min, and incubated at −20 °C for 1 h. The supernatant was removed by centrifugation at 12,000 rpm at 4 °C for 15 min. Then, the extract was dried in a vacuum concentrator, and 160 μL (acetonitrile to water volume ratio 1:1) was added to the dried metabolites for resolution, followed by a vortex for 30 s, ultrasonication in an ice water bath for 10 min, and centrifugation at 12,000 rpm, at 4 °C for 15 min. Finally, 120 μL of supernatant was removed, and 10 μL of each sample was subjected to quality control (QC) testing [54]. The LC/MS system for the metabolomics analysis consisted of Waters Acquity I-Class PLUS ultra-high performance liquid tandem Waters Xevo G2-XS QT of a high-resolution mass spectrometer (Milford, MA, USA). The column was purchased from Waters Acquity UPLC HSS T3 column (1.8 μm 2.1 × 100 mm). Positive ion mode: mobile phase A: 0.1% formic acid aqueous solution; mobile phase B: 0.1% formic acid acetonitrile; Negative ion mode: mobile phase A: 0.1% formic acid aqueous solution; mobile phase. B: 0.1% formic acid acetonitrile. The injection volume was 1 μL.

### 4.5. LC-MS/MS Analysis

Waters Xevo G2-XS QTOF high-resolution mass spectrometer collected primary and secondary mass spectrometry data in MSe mode under the control of the acquisition software (MassLynx V4.2, Waters). In each data acquisition cycle, dual-channel data acquisition could be performed on both low- and high-collision energy simultaneously. The low-collision energy is 2 V, the high-collision energy range is 10–40 V, and the scanning frequency is 0.2 s for a mass spectrum. The parameters of the ESI ion source are as follows: Capillary voltage: 2000 V (positive ion mode) or −1500V (negative ion mode); cone voltage: 30 V; ion source temperature: 150 °C; dissolvent gas temperature: 500 °C; backflush gas flow rate: 50 L/h; desolventizing gas flow rate: 800 L/h [55].

### 4.6. Western Blot

Cells from one confluent well of a six-well plate were lysed by adding RIPA lysis buffer supplemented with a protease inhibitor cocktail in an ice bath for 30 min. The lysates were centrifuged at 12,000× *g* for 10min at 4°C, and the supernatants containing total proteins were prepared. Then, 6 × loading buffer was added into the supernatant and placed in a 95 ℃ water bath for denaturation for 10 min. Moreover, the denatured total cell lysates were separated by 4–20% BisTris SDS-PAGE and transferred to PVDF membranes (Millipore). The membranes were blocked in 5% non-fat dry milk and inoculated with the following antibodies overnight: anti-HSV1 ICP0 (Cat. No. Ab6513, Abcam), anti-HSV1 + HSV2 ICP5 (Cat. No. Ab6508, Abcam), anti-GAPDH (Cat. No. Ag0766, Proteintech), anti-HSV1/2 ICP27 (Cat. No. sc-69806, SANTA CRUZ BIOTECHNOLOGY). The membranes were washed four times with TBS + 0.1%Tween-20. The membranes were incubated with horseradish peroxidase (HRP)-conjugated secondary antibodies (goat anti-mouse, Cat. No. SA00001-1, Proteintech) and developed using enhanced chemiluminescence (ECL) substrate. The results were confirmed by at least three biological replicates.

### 4.7. 50% Cell Culture Infectious Dose

Vero cells were evenly mixed with fresh DMEM containing 4% FBS and seeded in a 96 well plate with about 5 × 10^4^ cells per well. Then, the collected infectious supernatant was diluted ten-fold in fresh serum-free DMEM medium and added to the 96 well plate seeded with Vero cells for incubation for 5 days. Each dilution was tested with eight replicates for each experiment, and the wells were observed and scored for the presence or absence of cytopathic effect (CPE). Then, 50% cell culture infectious dose (CCID50) values were calculated using the Karber method [56].

### 4.8. Data Processing and Statistical Analysis

The raw data collected using MassLynx V4.2 were processed using the Progenesis QI software for peak extraction, peak alignment, and other data processing operations were identified based on the online METLIN database and Biomark self-built database. Moreover, the theoretical fragment identification and mass deviation were within 100 ppm [55]. The follow-up analysis was performed after normalizing the original peak area information with the total peak area. A principal component analysis (PCA) and Spearman’s correlation analysis were used to judge the repeatability of the samples within the group and the QC samples. The identified compounds were classified according to the pathway information in the Kyoto Encyclopedia of the Genes and Genomes (KEGG), the human metabolome database (HMDB), and lipid maps databases [57,58,59]. According to the grouping information, the difference multiples were calculated and compared, and a *t*-test was used to calculate the significant differences as the *p* value of each compound. The R language package ropls was used for orthogonal partial least squares discriminant analysis (OPLS-DA) modeling, and 200 times permutation tests were performed to verify the reliability of the model. The variable importance in the projection (VIP) value of the model was calculated using multiple cross-validations. The method of combining the difference multiples, the *p* value, and the VIP value of the OPLS-DA model was adopted to screen the differential metabolites. The screening criteria are a *p* value < 0.05 and VIP > 1. The difference metabolites of the KEGG pathway enrichment significance were evaluated using the hypergeometric distribution test [60].

## Figures and Tables

**Table 1:** The top 10 differential metabolites in the positive and negative ion modes, respectively, in descending order of VIP value. VIP is the VIP value of OPLS-DA model, *p* value is the *p* value of *t*-test.

#ID	Name	*p* Value	VIP	Regulated	Ion Mode
pos_6837	LysoPA(0:0/16:0)	1.46219 × 10^−6^	1.488614247	down	Positive
pos_759	2-Aminoheptanoate	1.37721 × 10^−5^	1.488078963	down	Positive
pos_7776	(4E)-3-Hydroxyhex-4-enoylcarnitine	0.000345637	1.487588232	down	Positive
pos_666	4-ethylamino-6-isopropylamino-1,3,5-triazin-2-ol	8.167 × 10^−7^	1.487449652	down	Positive
pos_3992	M8-Nelfinavir	1.19749 × 10^−5^	1.487062075	down	Positive
pos_7589	1-docosanoyl-glycero-3-phosphate	5.46165 × 10^−5^	1.487037578	down	Positive
pos_723	2-Quinoxalinol, 3-methyl-, 2-formate	0.000119334	1.486863687	down	Positive
pos_900	Isoamyl p-anisate	1.03039 × 10^−5^	1.486803732	down	Positive
pos_3937	Pro Ile Phe Met	2.33856 × 10^−6^	1.486218021	down	Positive
pos_7742	enantio-PAF C-16	0.000432337	1.486069983	down	Positive
neg_1917	Metoprolol acid	5.22685 × 10^−6^	1.330713177	down	Negative
neg_4481	Fluocortolone Pivalate	8.81614 × 10^−6^	1.330475128	down	Negative
neg_4581	Mopidralazine	5.94484 × 10^−5^	1.330473045	down	Negative
neg_4515	CDP-DG(i-16:0/a-25:0)	0.000129122	1.33012927	down	Negative
neg_4660	Cyclo(D-Trp-D-Asp-Pro-D-Ile-Leu)	8.30633 × 10^−6^	1.329777895	down	Negative
neg_4914	Dobutamine	8.13241 × 10^−5^	1.32974848	down	Negative
neg_3597	Leukotriene D4	1.26355 × 10^−5^	1.329743744	up	Negative
neg_4286	Palbociclib	1.5559 × 10^−5^	1.329483471	down	Negative
neg_4559	LysoPE(16:0/0:0)	1.62966 × 10^−5^	1.329429851	down	Negative
neg_4491	LysoPE(0:0/16:0)	3.95243 × 10^−5^	1.329411651	down	Negative

## Data Availability

The data presented in this study are available on request from the corresponding author.

## Supplementary Materials

The following supporting information can be downloaded at: https://www.mdpi.com/article/10.3390/ijms24021521/s1.
